# Coding and Noncoding Genes Involved in Atrophy and Compensatory Muscle Growth in Nile Tilapia

**DOI:** 10.3390/cells11162504

**Published:** 2022-08-12

**Authors:** Ali Ali, Walaa M. Shaalan, Rafet Al-Tobasei, Mohamed Salem

**Affiliations:** 1 Department of Animal and Avian Sciences, University of Maryland, College Park, MD 20742, USA; 2 Department of Zoology, Faculty of Science, Benha University, Benha 13518, Egypt; 3 Department of Biology, Middle Tennessee State University, Murfreesboro, TN 37132, USA; 4 Computational Science Program, Middle Tennessee State University, Murfreesboro, TN 37132, USA

**Keywords:** tilapia, fasting-refeeding schedule, muscle atrophy, compensatory growth, hypertrophy

## Abstract

Improvements in growth-related traits reduce fish time and production costs to reach market size. Feed deprivation and refeeding cycles have been introduced to maximize aquaculture profits through compensatory growth. However, the molecular compensatory growth signature is still uncertain in Nile tilapia. In this study, fish were subjected to two weeks of fasting followed by two weeks of refeeding. The growth curve in refed tilapia was suggestive of a partial compensatory response. Transcriptome profiling of starved and refed fish was conducted to identify genes regulating muscle atrophy and compensatory growth. Pairwise comparisons revealed 5009 and 478 differentially expressed (differential) transcripts during muscle atrophy and recovery, respectively. Muscle atrophy appears to be mediated by the ubiquitin-proteasome and autophagy/lysosome systems. Autophagy-related 2A, F-box and WD repeat domain containing 7, F-box only protein 32, miR-137, and miR-153 showed exceptional high expression suggesting them as master regulators of muscle atrophy. On the other hand, the muscle compensatory growth response appears to be mediated by the continuous stimulation of muscle hypertrophy which exceeded normal levels found in control fish. For instance, genes promoting ribosome biogenesis or enhancing the efficiency of translational machinery were upregulated in compensatory muscle growth. Additionally, myogenic microRNAs (e.g., miR-1 and miR-206), and hypertrophy-associated microRNAs (e.g., miR-27a-3p, miR-29c, and miR-29c) were reciprocally expressed to favor hypertrophy during muscle recovery. Overall, the present study provided insights into the molecular mechanisms regulating muscle mass in fish. The study pinpoints extensive growth-related gene networks that could be used to inform breeding programs and also serve as valuable genomic resources for future mechanistic studies.

## 1. Introduction

The skeletal muscle of fish comprises up to 60% of the body weight and provides proteins with low saturated fat and cholesterol content. Anabolic and catabolic pathways control muscle growth and atrophy [1]. Anabolic processes such as proliferation, differentiation, and fusion of myogenic precursor cells into myotubes (hyperplasia), as well as enlargement of pre-existing myofibres (hypertrophy), are responsible for the increase in mammalian muscle mass during embryonic/fetal and postnatal development, respectively [2]. In contrast to mammals, fish can exhibit intermediate growth in which hyperplasia and hypertrophy increase the body size and accumulate muscle throughout the entire life cycle [3,4]. Catabolic processes are regulated by several proteolytic systems, such as the autophagy/lysosome system, the ubiquitin-proteasome system, and calpains, which coordinate to degrade the muscle proteins [2]. Improving growth performance by reducing protein turnover and accelerating muscle protein accumulation requires understanding muscle plasticity in response to intrinsic and extrinsic factors [1].

Food availability is a major extrinsic factor influencing muscle plasticity [1]. Feed deprivation and refeeding protocols have been developed to maximize growth rates and aquaculture profits [5,6]. Feed-restricted fish can exhibit improved growth rates when subsequently provided with feed, a phenomenon called compensatory growth [7]. Compensatory growth could be an efficient management tool to improve growth rates [5,8]. Inducing compensatory growth in various aquaculture species improves feed utilization due to improved retention efficiency of ingested protein [9,10,11]. However, the compensatory gain capacity of tilapia is controversial. For example, complete compensation was reported in some studies, where the starved fish achieved the same weight as the fed fish [5,12]. By contrast, other studies have indicated partial compensatory capacity in Nile tilapia, meaning that feed-restricted fish can increase body weight during refeeding, but cannot reach the weight of continuously fed fish [13,14,15]. The physiological processes regulating the compensatory response are not well understood [16] and can be influenced by the periods of fasting and refeeding, species, age, sex, energy reserves, and environmental factors [5,7,12].

Compensatory growth has been widely observed in many teleost species, including Nile tilapia [15,17], Atlantic salmon [18], rainbow trout [9], channel catfish [19], gibel carp [20], tongue sole [21], army fish [22], European minnow [23], and barramundi [24]. However, the molecular mechanisms underlying the exaggerated growth phenotype remain scarce. Some studies have described the global gene expression patterns, during the fasting-refeeding schedule, in the skeletal muscle of rainbow trout [25], fine flounder [26], army fish [22], and grass carp [27]. Although these studies revealed changes in the transcriptome profile and identified genes potentially associated with compensatory growth, the upstream drivers modulating the global gene expression associated with compensatory growth in fish are still uncertain. None of the previous studies elucidated the regulatory role of microRNAs and long noncoding RNAs (lncRNAs) during compensatory growth. A more robust, holistic approach is needed to identify regulatory networks contributing to compensatory growth in fish.

MicroRNAs constitute a class of conserved, noncoding RNAs that negatively control gene expression at the post-transcriptional level [28]. Some microRNAs are mainly expressed in muscle and have crucial roles in skeletal muscle differentiation, development and atrophy such as miR-1a [29,30], miR-18a [31], miR-23a [32], miR-27b [33], miR-29b [34], miR-133a [29,30], miR-133b [35], miR-186 [36], and miR-206 [37]. For instance, miR-1 promotes myoblast differentiation and regeneration [29,30,38], whereas miR-29b is associated with loss of muscle mass [34]. Recent studies have established that microRNAs are involved in regulating muscle plasticity in fish. Tilapia miR-206, miR-203b and miR-181a-5p are involved in the negative regulation of IGF-1 [39], MyoD [40], and myostatin-b [41] expression, respectively. Differences in expression of miRNAs, involved in the GH/IGF-1 axis signaling pathway between tilapia fish with contrasting growth phenotypes suggest variation in miRNA expression as a mechanism affecting the capacity for muscle growth [42]. In tilapia muscle, changes in miR-125a-3p expression mediate regulation of the key regulator of branched-chain amino acid metabolism, Kruppel-like factor-15 (Klf15), during starvation [43]. Furthermore, seven selected miRNAs were found regulated in the skeletal muscle of grass carp within 1 or 3 h after refeeding [44]. However, feed restriction-refeeding studies in fish did not provide a comprehensive perspective of microRNA roles in skeletal muscle atrophy and/or recovery.

Similar to microRNAs, lncRNAs have demonstrated roles in regulating multiple biological processes, such as skeletal muscle differentiation and muscular atrophy. LncRNAs can regulate gene expression via direct interaction with microRNAs, mRNAs, and proteins [28,45]. For instance, long noncoding RNA, Muscle Differentiation 1 (lincMD1) regulates the time of muscle differentiation by acting as a competing endogenous RNA (ceRNA) in mouse and human myoblasts [46]. Linc-MD1 competes with MAML1 and MEF2C to sponge mir-135 and mir-133, respectively [46]. Myogenesis-associated lncRNA (lnc-mg) is a noncoding muscle differentiation and development regulator. Lnc-mg sponges miRNA-125b to regulate protein abundance of insulin-like growth factor 2 [47]. LncRNAs can also initiate the pathophysiological process of muscle wasting. For instance, Atrolnc-1 knockdown attenuates muscle wasting in mice with chronic kidney disease by inhibiting NF-κB activity and MuRF-1 [48]. Although muscle development and wasting mechanisms involve lncRNAs, the role of lncRNAs during skeletal muscle anabolic and catabolic states remains uncertain in fish.

In this study, fish were subjected to a two-week cycle of fasting-refeeding schedule. Partial compensatory capacity was achieved when fish were refed for two weeks following feed deprivation. To identify coding and noncoding genes involved in muscle atrophy and compensatory growth, we sequenced mRNAs, lncRNAs, and microRNAs from fish subjected to a fasting-refeeding protocol. We subsequently performed differential gene expression between fasting/fed and refed/control groups. Additionally, we explored functional interactions between differential transcripts (mRNAs, lncRNAs, and microRNAs) in terms of expression correlation and physical interaction to identify gene-regulatory circuits during starvation and compensatory growth. Our findings enhance our understanding of the mechanisms and identify growth-related gene networks underlying muscle atrophy and compensatory growth in Nile tilapia.

## 2. Materials and Methods

### 2.1. Ethics Declarations

The experiment was approved by the MTSU institutional animal care and use committee (IACUC), protocol 17-3008. All methods were carried out following relevant guidelines and regulations. The study was carried out in compliance with the ARRIVE guidelines (Animal Research: Reporting of In Vivo Experiments) [49].

### 2.2. Experimental Design

This experiment was previously described in [50]. In brief, tilapia fingerlings (Avg weight of 1.4 g) were obtained from Allin’s company, Chianti, CT, USA. Fish were acclimated for 15 days and then placed in 6 tanks (18 fish/tank). Tanks (50.5 × 25.5 × 31.5 cm) were equally divided into test and control groups. Fish from the test group were starved for two weeks and then manually fed a commercial fish diet (Allin’s company) for two more weeks. In contrast, fish from the control group were continuously fed for four weeks. Water quality parameters were maintained at temperature = 28–30 °C, pH = 7–7.2, ammonia = 0–0.25, nitrite = 0, and nitrate = 0. The two-week starved and refed fish and fed (2 weeks) and control fish (4 weeks) were euthanized using an overdose of MS222. Muscle samples were flash-frozen in liquid nitrogen and stored at −80 °C.

### 2.3. RNA Extraction, Library Preparation, and Sequencing

RNA was extracted from the muscle of 32 fish (9 fed, 9 control, 8 starved, and 6 refed) by using TriZol (Invitrogen, Carlsbad, CA, USA), followed by quantity and quality assessments. Library preparation was carried out according to the service provider’s instructions (BGI Americas Corporation, Cambridge, MA, USA). Total RNA was treated with DNase I to degrade DNA contamination, if any. Then mRNA was enriched using the oligo (dT) magnetic beads. The mRNA was fragmented and then a random hexamer-primer was used to synthesize the first strand of cDNA. The second strand was synthesized followed by end repair, 3′-end single nucleotide A (adenine) addition, and adapter ligation. The fragments were enriched by PCR amplification. Finally, single strand separation, cyclization, and DNA nanoball synthesis were performed. The library products were sequenced on a DNBseq platform. For microRNA sequencing, 24 libraries (6 libraries/condition) were prepared following the BGI’s instructions and then sequenced on a DNBseq platform.

### 2.4. Prediction of Novel lncRNAs

LncRNAs were identified from sequencing reads by modifying the pipeline we previously described [51]. In Brief, reads were mapped to the Nile tilapia reference genome using TopHat (Toronto, ON, Canada) and HISAT2 and assembled using Cufflinks and StringTie, respectively. Transcripts shorter than 200 nucleotides and transcripts showing similarity to protein-coding genes with E-value < 10^−5^ were filtered out. Transcripts were checked out for predicted protein-coding potential (CPC score > −1) or similarity to protein-coding domains in Pfam. In addition, BlastN was used to filter out transcripts that have any match with other noncoding RNA families. Putative lncRNA transcripts from the assembly are available at https://osf.io/by64h/ (accessed on 19 May 2022).

### 2.5. Differential Gene Expression Analyses

The tilapia Ensembl annotation file was downloaded and modified to include the novel lncRNAs identified in this study. Read mapping was performed using the CLC genomics workbench. Raw counts were used to identify differentially expressed protein-coding transcripts and lncRNAs using DESeq2 (Padj-value < 0.05, fold change ≥ |2|).

For microRNAs, reads were mapped to the miRBase (Release 22.1), and the four species *Oreochromis niloticus*, *Danio rerio*, *Oryzias latipes*, and *Homo sapiens* were prioritized. Additionally, Ensembl noncoding RNAs of *Oreochromis niloticus*, *Danio rerio*, *Oreochromis aureus*, *Oryzias latipes*, and *Homo sapiens* were included for the mapping. The CLC genomics workbench mapping criteria allowed maximum mismatch = 2, and additional/missing upstream/downstream bases = 2. The total read counts were normalized using the TMM normalization method to identify differential microRNAs (FDR-*p*-value < 0.05, fold change ≥ |2|).

### 2.6. MicroRNA Target Prediction and Functional Enrichment Analyses

Transcripts with a fold change ≥ 4 or ≤ 4 were used for the microRNA target prediction analysis. MicroRNA binding sites were computationally predicted in the 3′ UTR of differential mRNAs, whereas microRNA target sites were searched throughout the entire lncRNA sequences. MiRanda and PITA were run through the sRNAtoolbox to find the target genes. Target sites predicted by both prediction algorithms were considered potential microRNA binding sites.

Functional enrichment analyses for differential protein-coding transcripts and microRNA target transcripts were performed by using g:Profiler (adj *p*-value < 0.05) [52].

## 3. Results

### 3.1. Fish Compensatory Growth Following Fasting-Refeeding Schedule

**Figure 1:**
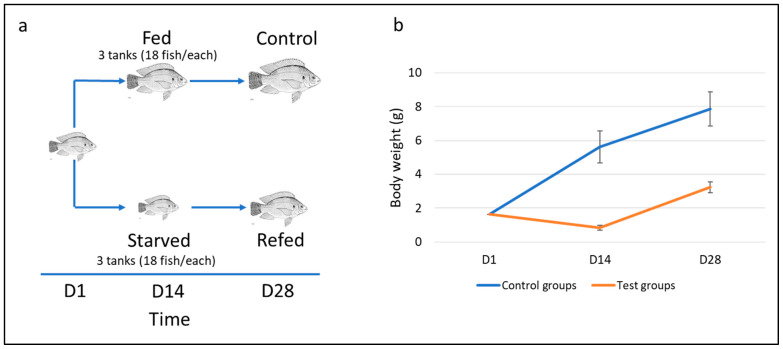
(**a**) Illustration of the experimental design. Fish were subjected to a two-week cycle of fasting-refeeding schedule. (**b**) Changes in tilapia body weight over the time course of the fasting-refeeding experiment. Test groups include the starved group (D14) and refed group (D28), whereas control groups include the fed group (D14) and control group (D28).

Fish were subjected to a two-week fasting-refeeding schedule, as shown in Figure 1a. Following 14 days of feed restriction, the body weight of starved fish (0.839* ± 0.154 g) decreased significantly compared to that of the fed group (5.621 ± 0.942 g; *p* < 0.05). After 14 days of refeeding, a remarkable increase in the body weight of refed fish was observed (Figure 1b). Notably, the refed fish exhibited an average body weight gain of 2.40 g, slightly higher than that of the control group (2.26 g). These results suggest partial compensatory capacity was achieved when fish were refed for two weeks following feed deprivation. Longer refeeding time is likely needed to achieve complete compensation.

### 3.2. Muscle Transcriptome Sequencing and Data Processing

**Figure 2:**
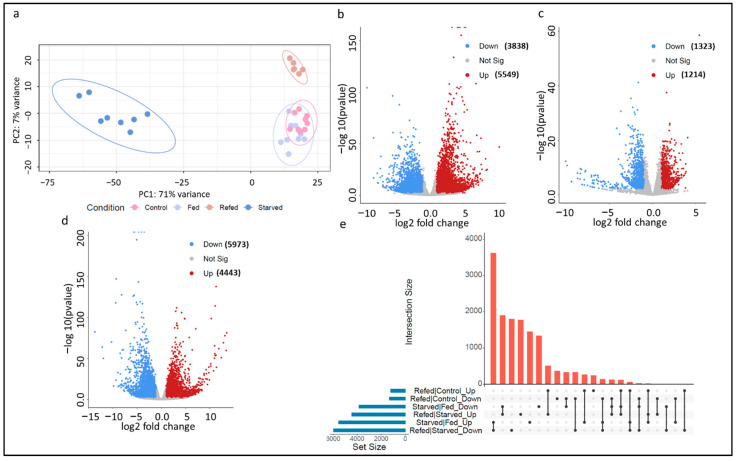
(**a**) Principal component analysis of tilapia transcripts obtained from 32 RNA-seq datasets generated from skeletal muscle of starved, fed, refed, and control fish. Each round dot represents a single RNA-seq dataset color-coded by treatment group. (**b**–**d**) Volcano plots showing the tilapia transcripts (mRNAs, lncRNAs, and microRNAs) differentially expressed between starved/fed, refed/control, and refed/starved groups. The red dots represent upregulated transcripts, whereas the blue dots represent downregulated transcripts at FDR < 0.05 and fold change > |2|. (**e**) UpSet chart showing the overlap among differential transcript sets obtained from the three pairwise comparisons. The horizontal bar plot represents the set sizes. The dot plot represents the set participation in the intersection, whereas the vertical bar plot represents the intersection size.

To identify genes contributing to skeletal muscle atrophy and growth compensation, we sequenced mRNAs, lncRNAs, and microRNAs in the muscle of starved, fed, refed, and control fish. A total of 1,559,142,712 RNA sequence reads were produced (Avg. 49 M reads per sample) from 32 libraries (see Methods section). We first sought to improve the tilapia lncRNA reference by genome-wide identification of novel lncRNA transcripts using the RNA-Seq datasets generated in this study. In total, 6330 novel lncRNA transcripts were identified. Novel lncRNAs were merged with the Ensembl genome reference annotation, which was used as a reference for differential gene expression analyses (Figure 2b–e). Notably, 92% of the reads were mapped to the tilapia reference assembly. A principal component analysis showed a clear separation of the starved fish from other groups. Refed samples were clustered separately from the control and fed samples (Figure 2a).

Furthermore, high throughput small RNA sequencing from 24 libraries resulted in a mean sequencing depth of ~11.5 million reads per sample. The average number of reads produced from each sample was 21,829,322 (Avg. length of 22 nucleotides). On average, 143,763 potential microRNAs were detected in each sample. Of these potential microRNAs, ~22% had sequence homology with the miRBase mature microRNAs. MicroRNAs were clustered according to sequence homology into 3636 unique mature microRNAs and used for differential expression analysis between starved/fed (Figure 2b), refed/control (Figure 2c), and refed/starved (Figure 2d) groups.

### 3.3. Muscle Atrophy Mediated by Ubiquitin-Proteasome and Autophagy-Lysosome Systems Accompanied by Maintenance of the Nervous System

To identify genes likely contributing to skeletal muscle atrophy during starvation, we performed differential gene expression analysis between atrophying and non-atrophying skeletal muscles collected from starved and fed groups, respectively. A total of 8930 mRNAs, 287 lncRNAs, and 170 microRNAs were differential between fish groups (FDR *p*-value < 0.05, fold change: > |2|) (Figure 2b and Supplementary Table S1). Of them, 5549 transcripts (5279 mRNAs, 146 lncRNAs, and 124 microRNAs) were upregulated, and 3838 transcripts (3651 mRNAs, 141 lncRNAs, and 46 microRNAs) were downregulated in the starved compared to fed fish (Figure 2b). Several differential transcripts were selected for validation by qPCR (Table 1).

**Figure 3:**
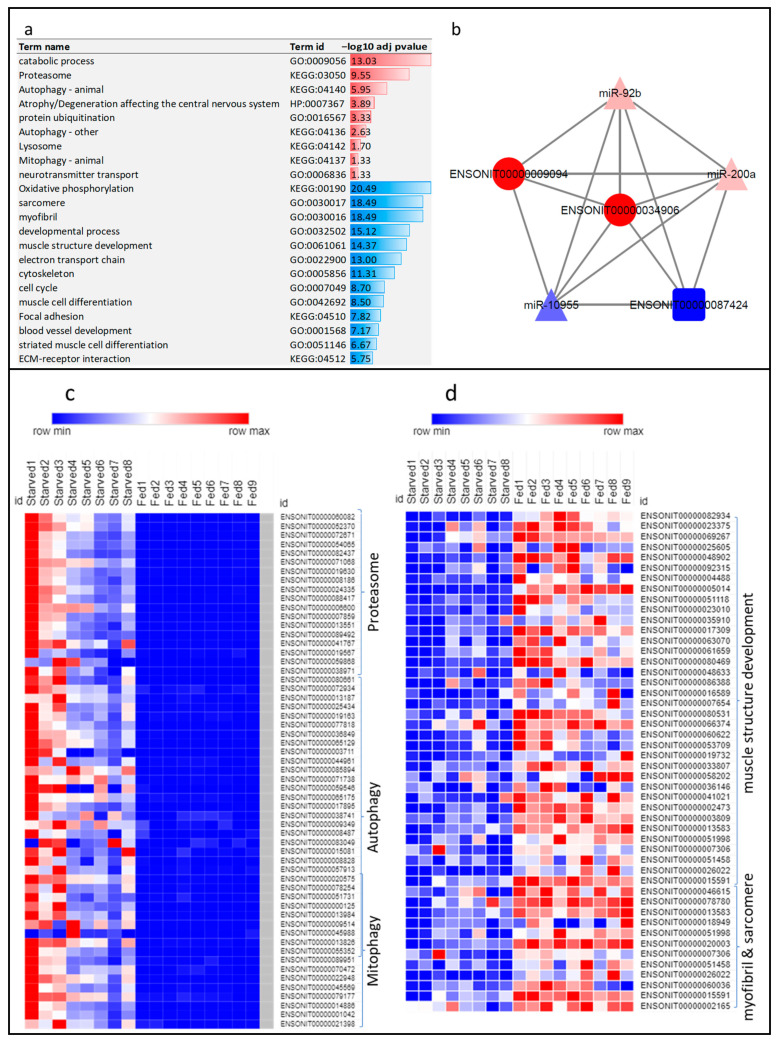
(**a**) Enrichment analysis of differential transcripts following starvation. Over-represented KEGG pathways and GO terms are represented in red, whereas under-represented KEGG pathways and GO terms are represented in blue. Negative log10 of adj *p*-values are plotted. (**b**) Interaction network between FBXO32 transcripts and noncoding transcripts (lncRNA and microRNAs) exhibiting correlation in expression. FBXO32 transcripts and the lncRNA ENSONIT00000087424 share similar microRNA binding sites. (**c**) Heatmap showing expression pattern of starvation-upregulated transcripts involved in proteasome, autophagy, and mitophagy (fold change > 4). (**d**) Heatmap showing expression pattern of starvation-downregulated transcripts involved in muscle structure development and myofibril and sarcomere (fold change < −4).

Genes promoting proteolysis/catabolic processes were significantly upregulated in atrophying skeletal muscle of starved fish (Figure 3a). A total of 125 transcripts involved in protein ubiquitination, 56 transcripts relevant to the proteasome, 127 transcripts involved in autophagy/lysosome, and 24 transcripts involved in mitophagy showed upregulation in atrophying muscle (Figure 3a,c). On the other hand, coding transcripts that negatively regulate the ubiquitin-proteasome system (ubiquitin-like domain-containing CTD phosphatase 1) were downregulated.

Consistent with our previous study on rainbow trout [28], F-box-only protein 32 (FBXO32) was among the most upregulated genes suggesting a major proteolytic role during skeletal muscle atrophy. FBXO32 transcripts, ENSONIT00000034906 and ENSONIT00000009094, exhibited 103- and 80-fold change, respectively (Supplementary Table S1). Consistent with the RNA-Seq results, the FBXO32 showed upregulated expression by quantitative PCR in starved tilapia (Table 1). FBXO32 transcripts exhibited expression correlation and shared miR-92b, miR200a, and miR-10955 binding sites with the lncRNA ENSONIT00000087424 (act as a potential microRNA sponge) (Figure 3b). Also, FBXO32 exhibited a negative correlation (R = −0.69; *p*-value 9.83 × 10^−5^) with the body weight regardless of the treatment group (Table 1).

Oxidative phosphorylation, cell cycle, and actin cytoskeleton regulating genes were downregulated, while genes involved in fatty acid degradation were mainly upregulated. Similarly, genes associated with myofibril, sarcomere, muscle structure development, muscle contraction, and extracellular matrix were downregulated and significantly underrepresented (Figure 3a,d), consistent with the muscle mass loss during atrophy. For instance, transcripts encoding collagen (n = 60), troponin (n = 32), and myosin, myosin-binding protein and tropomyosin (n = 67) were significantly downregulated. In addition, 47 extracellular matrix proteins were downregulated. Our previous study also revealed similar gene expression patterns in atrophying skeletal muscle of rainbow trout during sexual maturation [28]. These findings suggest that muscle degradation in fish is driven by the upregulation of ubiquitin-proteasome and autophagy-lysosome systems with concomitant downregulation of genes encoding muscle sarcomere and extracellular matrix proteins.

Simultaneously, genes involved in the nervous system development were upregulated and significantly overrepresented in response to starvation (Figure 3a). For instance, neurofilament, light polypeptide b (NEFL) has dramatically increased following feed restriction (Table 1). The neurodegenerative biomarker NEFL increases in response to axonal damage [53]. Also, starved fish upregulated other transcripts involved in axon development, such as dihydropyrimidinase-related protein 5 (DPYSL5) [54] and atlastin GTPase 1 (ATL1) [55].

**Figure 4:**
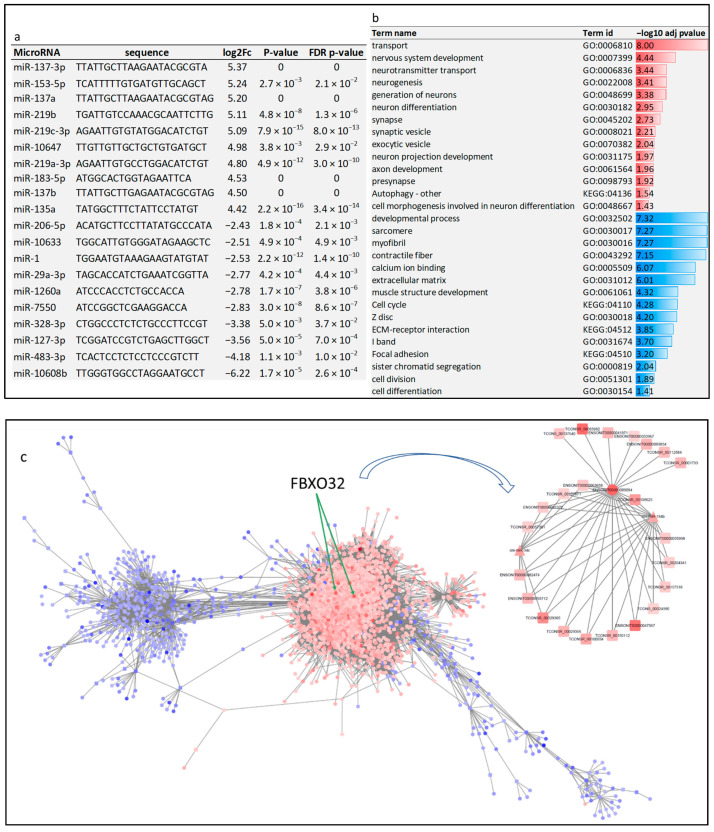
(**a**) A selected set of the most significantly differential microRNAs in atrophying muscle of starved versus fed fish. MicroRNA-137-3p was the most highly expressed, and miR-10608b was the most downregulated microRNA due to feed restriction. Several isoforms of miR-137 were upregulated in starved fish. Fold change was considered significant at cutoff value >2 or <−2 and FDR *p*-value < 0.05. (**b**) Enrichment analysis of differential transcripts potentially targeted by differential microRNAs following starvation. Over-represented KEGG pathways and GO terms are represented in red, whereas under-represented KEGG pathways and GO terms are represented in blue. Negative log10 of adj *p*-values are plotted. (**c**) Gene expression network of differential mRNAs (circular nodes), lncRNAs (rectangular nodes), and microRNAs (triangular nodes) (R > 0.80 or <−0.80). The red nodes represent upregulated transcripts in the starved fish (fold change > 4), whereas the blue nodes represent downregulated transcripts (fold change < −4) at FDR ≤ 0.05. Most of the differential transcripts are clustered in two major networks. The large network (tilapia degradome) mainly includes upregulated transcripts, whereas the small network includes downregulated transcripts in response to feed restriction. Two upregulated FBXO32 transcripts appear in the center of the large network. Nodes (transcripts) are connected by edges representing the expression correlation. The shorter the edge’s length, the stronger the expression correlation.

A total of 170 microRNAs were differential in the skeletal muscle of starved compared to fed fish (Supplementary Table S1). The top upregulated and downregulated microRNAs are provided in Figure 4a. Most of the differential microRNAs (n = 124) were upregulated in response to starvation. Notably, mir-1 and mir-133 were downregulated in the atrophying tilapia muscle, consistent with other fish and mammalian species studies [28,56,57]. A total of 2424 differential mRNA transcripts were computationally predicted as target genes of the differential microRNAs (log2FC > 2). differential microRNAs and ~64% of their predicted differential mRNA targets (n = 1562) showed reciprocal differential expression (Supplementary Table S2). Forty-six downregulated microRNAs were potentially targeting 788 unique protein-coding transcripts. Gene set enrichment analysis revealed that these target transcripts belong to the autophagy pathway (n = 5), nervous system development (n = 54), and neurotransmitter transport (n = 10) (Figure 4b). Autophagy-related proteins such as Atg9a and ATG4B have neuroprotective functions [58,59]. Eighty-five upregulated microRNAs (log2FC > 2) were predicted to target 774 unique protein-coding transcripts. Consistent with the fact of muscle loss during starvation, functional enrichment analysis revealed that these coding transcripts are annotated to developmental process (n = 137), cell cycle (n = 34), muscle structure development (n = 22), myofibril (n = 13), sarcomere (n = 13), and extracellular matrix (n = 14) (Figure 4b). These findings suggest that the fate of some of the genes involved in skeletal muscle atrophy is post-transcriptionally determined by altered expression of a group of microRNAs.

Oni-miR-10608b was the most highly downregulated microRNA (−75 fold) in response to starvation (Figure 4a and Supplementary Table S1). This microRNA has recently been identified in tilapia [60]. It was predicted to target 85 protein-coding transcripts, which exhibited reciprocal differential expression (Supplementary Table S2). Coding transcripts targeted by Oni-miR-10608b with the highest upregulation are mainly involved in maintaining the nervous system structure and function, including stathmin-2 [61], complexin-2 [62], and neural EGFL like 2 [63]. Consistent with the oni-miR-10608b downregulation in atrophying muscle, some target transcripts of proteolytic functions were identified, such as F-box/WD repeat-containing protein 11, proteasome 20S subunit alpha 4, and ubiquilin 4. Of note, 16 lncRNA transcripts were predicted to sponge the oni-miR-10608b and release 93 protein-coding transcripts (e.g., neurexin 3b, neural EGFL 2, and complexin-2) from the microRNA control (Supplementary Table S3).

Conversely, miR-137-3p, miR-153-5p, and miR-137a were highly upregulated (~38.5 x fold) in atrophying skeletal muscle (Figure 4a and Supplementary Table S1). MicroRNA-137-3p and miR-137a were predicted to target 41 transcripts (Supplementary Table S2). Whereas, miR-153-5p was predicted to target 141 unique transcripts (Supplementary Table S2). Consistent with the upregulation of these microRNAs in atrophying muscle, predicted target transcripts were involved in the cell cycle (e.g., BUB1 mitotic checkpoint serine/threonine kinase B), Z disc (e.g., LIM domain-binding protein 3), structural constituents of muscle (e.g., myosin XVI, myomesin 1b, and tropomyosin 1), extracellular matrix (e.g., collagen, type VI, alpha 1), and developmental process (e.g., POU class 6 homeobox 2). These findings suggest that differential microRNAs likely contribute to muscle mass loss and nervous tissue maintenance following feed restriction.

### 3.4. ‘LncRNA-mRNA-microRNA’ Interactome in Atrophying Skeletal Muscle

We computed the lncRNA-mRNA-microRNA interaction networks during muscle atrophy based on their normalized expression pattern across 24 RNA-Seq datasets. The gene networks comprised 3839 differential transcripts at a correlation threshold of R > 0.80 or <−0.80. The differential transcripts in response to feed restriction were mainly clustered in two networks (Figure 4c). A large network includes mainly upregulated transcripts, about 81% of the regulated transcripts, and a small network includes downregulated transcripts. The large network consisted of 3118 transcripts (94 lncRNAs, 2946 mRNAs, and 78 microRNAs). In contrast, the small network consisted of 374 transcripts (25 lncRNAs and 349 mRNAs and no microRNAs). The large network appears to be the major driver regulating muscle proteolysis as the network consists mainly of proteolytic genes such as enzymes of the ubiquitin-proteasome system and autophagy- and mitophagy-related genes. Similarly, most of the upregulated microRNAs, such as miR-137-3p, miR-153-5p, and miR-137a, were a part of the large network. Two upregulated FBXO32 transcripts (the top upregulated ubiquitin proteins) and miR-137-3p (the top upregulated microRNA) appeared in the center of the large network suggesting a key role in muscle degradation. The FBXO32 was previously reported to play a crucial role in muscle atrophy in fish and other vertebrates [28,64]. Therefore, the large network functions as ‘the Nile tilapia muscle degradome’. Figure 4c shows a sub-network of the degradome consisting of FBXO32 transcripts and all interacting differential lncRNAs and microRNAs. Our results suggest that both coding and noncoding genes work together to coordinate the process of muscle atrophy in fish.

### 3.5. Suppression of Catabolic Processes and Induction of Muscle Hypertrophy upon Refeeding

**Figure 5:**
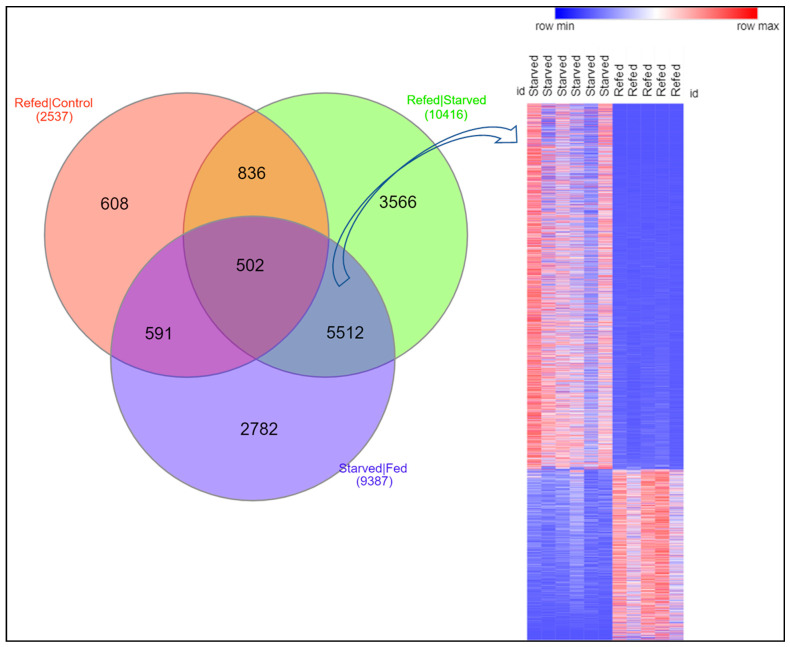
Venn diagram (**left**) showing the number of differential transcripts shared between different treatment groups, and heatmap (**right**) showing that refed fish reversed the expression of most of the differential transcripts in response to starvation. The expression pattern of fish sequenced for both total RNA and microRNAs (six starved and five refed) was displayed on the heatmap.

To identify genes potentially contributing to muscle growth compensation, we performed differential gene expression analysis between refed and control fish. A total of 2343 mRNAs, 71 lncRNAs, and 123 microRNAs were differential between both fish groups (FDR *p*-value < 0.05, fold change > |2|) (Figure 2c and Supplementary Table S1). Of them, 1214 transcripts (1165 mRNAs, 27 lncRNAs, and 22 microRNAs) were upregulated, and 1323 transcripts (1178 mRNAs, 44 lncRNAs, and 101 microRNAs) were downregulated in refed fish compared to the control group (Figure 2c). Many starvation-caused changes in the skeletal muscle transcriptome were reversed upon refeeding (Figure 5). Several differential transcripts were selected for validation during starvation and following refeeding (Table 1). Notably, these transcripts showed significant correlations with body weight (Table 1).

To provide insights into the transcriptomic changes occurring upon refeeding, differential transcripts in the refed group were classified into three clusters: (1) transcripts whose expression levels were restored to normal levels in the refed fish; (2) transcripts whose expression levels in refed fish exceeded that of control fish; and (3) transcripts whose expression levels in refed fish were less than those of control fish.

### 3.6. First Cluster: Transcripts Whose Expression Levels in Refed Fish Were Restored to Normal Levels

**Figure 6:**
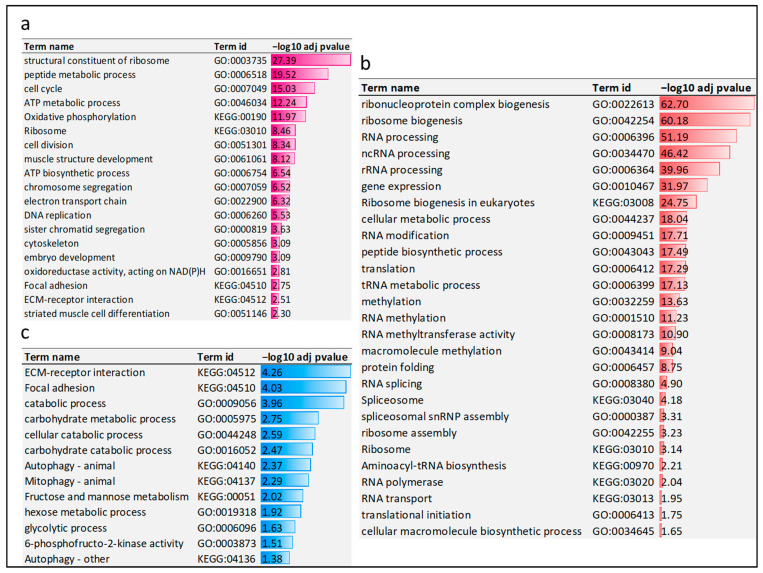
Functional enrichment analyses. (**a**) Enrichment analysis of transcripts whose expression levels in refed fish were restored to normal levels (first cluster). Enriched KEGG pathways and GO terms are represented in purple. (**b**) Enrichment analysis of transcripts whose expression levels in refed fish exceeded normal growth values (second cluster). Over-represented KEGG pathways and GO terms are represented in red. (**c**) Enrichment analysis of transcripts whose expression levels in refed fish did not reach normal growth values (third cluster). Under-represented KEGG pathways and GO terms are represented in blue. Negative log10 of adj *p*-values are plotted.

The first cluster comprises 3661 transcripts whose expression increased after refeeding to reach normal growth levels. The cluster encompasses 3515 mRNAs, 101 lncRNAs, and 45 microRNAs. Functional enrichment analysis was performed to provide insights into the functional significance of the protein-coding transcripts. The analysis showed enrichment of genes mapped to eight KEGG pathways such as oxidative phosphorylation (adj *p* < 1.06 × 10^−12^), cell cycle (adj *p* < 1.36 × 10^−5^), ribosome (3.43 × 10^−9^), and focal adhesion (adj *p* < 1.79 × 10^−3^) (Figure 6a). Additionally, genes in this cluster showed enrichment in biological GO terms linked to cell division (adj *p* < 4.52 × 10^−9^), chromosome segregation (adj *p* < 3.05 × 10^−7^), DNA replication (adj *p* < 2.97 × 10^−6^), muscle structure development (adj *p* < 7.56 × 10^−9^), embryo development (adj *p* < 8.13 × 10^−4^), striated muscle cell differentiation (adj *p* < 5.00 × 10^−3^), and cytoskeleton (adj *p* < 8.08 × 10^−4^) (Figure 6a). Consistent with our data, genes identified in this cluster overlapped with ~37% of the genes restored to normal levels during the rainbow trout’s fasting-induced recovery growth [65]. Gene enrichment analysis revealed that overlapping genes were enriched in biological GO terms linked to cell cycle (adj *p* < 6.78 × 10^−7^), cell division (adj *p* < 3.43 × 10^−4^), and chromosome condensation (adj *p* < 4.93 × 10^−2^). To provide insights into the function of lncRNAs restored to normal levels, we searched for their neighboring genes. We found 39 neighboring protein-coding genes enriched in positive regulation of transcription and protein oxidation.

The expression levels of 45 microRNAs were restored to their normal levels. The microRNA targets were enriched in biological GO terms linked to cell development (adj *p* < 6.07 × 10^−4^), anatomical structure morphogenesis (adj *p* < 4.73 × 10^−3^), anatomical structure development (adj *p* < 1.73 × 10^−2^), and cell differentiation (adj *p* < 4.38 × 10^−2^). The list included microRNAs known to function in skeletal myogenesis, such as miR-214, miR-206-5p, miR-221-5p, miR-222, miR-133b-3p, and miR-21 [66,67]. Together, our results show that refed fish have restored the normal expression levels of genes involved in cell proliferation and skeletal muscle cell differentiation.

### 3.7. Second Cluster: Transcripts Whose Expression Levels in Refed Fish Exceeded Normal Growth Values

The second cluster comprises 1214 upregulated transcripts (1165 mRNAs, 27 lncRNAs, and 22 microRNAs) in the refed fish compared to control tilapia. Genes in this cluster showed enrichment in biological terms associated with transcriptional and post-transcriptional processes such as ribonucleoprotein complex biogenesis (adj *p* < 1.99 × 10^−63^), RNA processing (adj *p* < 6.52 × 10^−52^), ncRNA processing (adj *p* < 3.81 × 10^−47^), and RNA splicing (adj *p* < 1.26 × 10^−5^) (Figure 6b). Other GO terms linked with genes in the second cluster included translation (adj *p* < 5.17 × 10^−18^), peptide biosynthetic process (adj *p* < 3.21 × 10^−18^), protein folding (adj *p* < 1.76 × 10^−9^), ribosome biogenesis (adj *p* < 6.59 × 10^−61^), and ribosome assembly (adj *p* < 5.83 × 10^−4^) (Figure 6b). Although ribosome-related terms were enriched in the first cluster, the terms linked to ribosome biogenesis and ribosome assembly weren’t entirely detected in the first cluster. The second cluster also included epigenetic regulators (37 transcripts involved in RNA modification/methylation). Among them were the histone-modifying enzymes of the protein arginine methyltransferase family, such as PRMT1, PRMT3, PRMT5, and PRMT7. Remarkably, genes identified in this cluster overlapped with genes expressed in the rainbow trout superficial hyperplastic growth zone [68] and during the trout compensatory muscle growth signature [65]. Common genes were related to spliceosome, histone modification, and protein biosynthesis. Myogenesis- and cell cycle-related genes which characterize the superficial hyperplastic growth zone were absent in this cluster. Loss or gain-of-function experiments revealed a list of 47 genes that can cause skeletal muscle hypertrophy in mice [69]; nine of them were identified in the tilapia compensatory growth response (Table 2). Two out of the nine genes were upregulated in refed fish. These genes encode JunB proto-oncogene and calpastatin, which induce muscle hypertrophy [70,71]. Four transcripts encoding calpain-3, calpain-5, calpain-9, and calpain-15 were downregulated upon refeeding.

MicroRNA-4585, miR-29d, miR-460, and miR-460-5p were highly upregulated in the refed fish (Supplementary Table S1). These microRNAs were predicted to target 24 protein-coding transcripts, which exhibited reciprocal differential expression (Supplementary Table S2). Consistent with the upregulation of these microRNAs in the refed fish, predicted target transcripts were mainly involved in catabolic processes. For instance, miR-4585 was predicted to target TSC22 domain family protein 3 (TSC22D3) (Supplementary Table S2), which inhibits myogenic differentiation [72]. Whereas miR-460 and miR-460-5p were predicted to target forkhead box protein O1-A, F-box only protein 32, and autophagy-related protein 9A (Supplementary Table S2). Furthermore, microRNAs involved in muscle hypertrophy were upregulated in the refed fish. Hypertrophy-associated microRNAs included miR-27a-3p, miR-29c, and miR-29c [73,74,75]. Our findings suggest these microRNAs are upstream drivers modulating the global gene expression associated with muscle growth following refeeding. Of note, 11 lncRNAs identified in the compensatory growth signature exist on the tilapia genome within less than 15Kb away from genes involved in the FoxO signaling pathway, suggesting a role for these lncRNAs in regulating tilapia muscle hypertrophy.

### 3.8. Third Cluster: Transcripts Whose Expression Levels in Refed Fish Were Less than Normal Growth Values

The third cluster comprises 1323 downregulated transcripts in the skeletal muscle of refed fish compared to control tilapia. The cluster encompasses 1178 mRNAs, 44 lncRNAs, and 101 microRNAs. This cluster complement the compensatory muscle growth signature identified in the second cluster. Genes promoting proteolysis/catabolic processes were significantly downregulated after refeeding. Twenty transcripts involved in autophagy and 12 transcripts involved in mitophagy showed downregulation in skeletal muscle during recovery. Additionally, 27 transcripts in this cluster showed enrichment in focal adhesion (adj *p* < 9.33 × 10^−5^) (Figure 6c). Transcripts involved in FoxO signaling pathway that were upregulated in response to starvation were also significantly under-represented upon refeeding. Consistently, our previous study revealed reciprocal differential expression of 10 catabolic genes, including FBXO32, in response to starvation and subsequent refeeding [50]. In this study, the reciprocal expression of several catabolic genes was also validated by qPCR (Table 1). Genes involved in FoxO signaling and focal adhesion pathways, such as FoxO1 [76], caveolin 1 [77], FYN proto-oncogene [78], and epidermal growth factor receptor [79], negatively regulate muscle hypertrophy.

In addition, the carbohydrate metabolic and glycolytic processes were among the significantly under-represented biological processes (Figure 6c). Reduced glycolysis was previously reported during the initial refeeding phase until glycogen levels are restored [80,81]. It is noteworthy that seven out of the 47 hypertrophy-associated genes reported in mice were identified in this cluster [69]. The list included transcripts encoding myostatin, tumor protein p53 inducible nuclear protein 2 (TP53INP2), and nuclear receptor corepressor 1 (NCoR1) (Table 1 and Table 2). These findings indicate that the compensatory muscle growth is driven by suppression of catabolic pathways alongside stimulation of muscle hypertrophy.

In contrast to starvation, most differential microRNAs (n = 101) were downregulated in the refed group. The most highly downregulated microRNAs in the refed fish were miR-485-5p (−1090 fold change) and miR-219a-2-3p (−975 fold change) (Supplementary Table S1). In this study, miR-485-5p was predicted to target 9 protein-coding transcripts, which exhibited reciprocal upregulated expression in the refed fish (Supplementary Table S2). Some target transcripts are essential in rRNA processing (e.g., TSR2 ribosome maturation factor “TSR2”), maintaining amino acids supply essential for protein production and energy transfer within the cell (e.g., proline dehydrogenase 1, mitochondrial “PRODH”) [82], maintaining the structure of sarcomeres (e.g., desmin), and enhancing fatty acid synthesis (e.g., stearoyl-CoA desaturase) [83]. Furthermore, synchronized downregulation of important muscle-relevant microRNAs was observed in the muscle of the refed tilapia including miR-1, miR-206, mir-10a, miR-139, miR-135b, miR-150, miR-185, and members from miR-30 family (miR-30a-5p, miR-30c-1-3p and miR-30c-2-3p). These findings suggest that microRNAs might play a regulatory role in mediating skeletal muscle compensatory response in Nile tilapia by stimulating muscle hypertrophy.

The second and third clusters show that fasting-induced muscle recovery occurs by enhancing the anabolic processes essential for protein synthesis and folding and suppressing catabolic processes such as autophagy and mitophagy. Overall, our study shows that hypertrophy is likely the major contributor to compensatory muscle growth in Nile tilapia.

## 4. Discussion and Conclusions

Starvation and compensatory growth occur in nature and aquaculture. Several fish species have reported a massive reduction in body weight due to starvation [5,12,22,65]. The compensatory growth phenomenon has previously been reported in other fish species, including Atlantic salmon [18], rainbow trout [9,65], channel catfish [19], gibel carp [20], tongue sole [21], army fish [22], European minnow [23], and barramundi [24]. In this study, Nile tilapia were starved for 14 days, followed by 14 days of refeeding. Our experiment was designed with extended fasting and short refeeding periods compared to other studies and allowed only partial compensatory growth after refeeding [22].

The molecular mechanism regulating muscle growth under feed restriction-refeeding conditions has not been fully elucidated in fish [22]. Many of the previous studies investigating fish muscle atrophy provided limited information since only a limited set of genes were investigated. The present study characterized coding genes, lncRNAs, and microRNAs potentially involved in Nile tilapia muscle atrophy. We investigated lncRNA-mRNA-microRNA interactions (muscle degradome) that likely regulate muscle proteolysis. differential lncRNA exhibited the potential to sequester/sponge microRNAs involved in muscle atrophy. In tilapia, starvation increased the expression of genes involved in proteasome and autophagy but reduced the expression of genes associated with oxidative phosphorylation and the cell cycle. These results are consistent with previous studies that demonstrated the crucial role of genes encoding the ubiquitin-proteasome system and autophagy-related proteases during muscle atrophy [28].

In fish, skeletal muscle growth can occur by hypertrophy (size increase) and hyperplasia (the genesis of new myofibres) [84,85]. Myogenic regulatory factors regulate myoblast proliferation and differentiation. The genetic mechanisms regulating the production of new myofibres have previously been described in fish. Rescan et al. profiled the transcriptome of the superficial hyperplastic growth zones of the myotome in late trout embryos [68]. The authors further characterized the molecular mechanism involved in the compensatory growth response in trout [65]. Comparing the molecular signature of the superficial hyperplastic growth zones with that of the compensatory growth revealed common genes involved in protein biosynthetic and maturation processes such as RNA processing, ribosome biogenesis, translation, protein folding, and ribonucleoprotein complex biogenesis [65]. To investigate the relative contribution of both hypertrophy and hyperplasia in the compensatory capacity of Nile tilapia, we performed a comprehensive transcriptomic analysis in the skeletal muscles of starved and refed fish. Gene expression profiling revealed regulated genes in refed fish clustered into three distinct categories: (1) genes whose expression level exceeded that of starved fish but restored to the values found in control tilapia; (2) genes whose expression exceeded that of control tilapia; and (3) genes whose expression levels in refed fish was less than normal growth values. Genes of the first category indicated the restoration of cellular processes contributing to normal growth. Whereas genes of the second and third categories likely contribute to the exaggerated growth phenotype. Genes whose expression was restored after refeeding have GO terms involved in DNA replication, cell cycle, muscle structure development, and striated muscle cell differentiation, suggesting a resumption of cell proliferation and differentiation to reach normal levels in refed fish. GO terms associated with DNA replication and cell cycle in hyperplastic growth zones [68] were absent in rainbow trout’s compensatory muscle growth signature [65].

Hyperplasia-associated genes coding for promyogenic membrane receptors [86,87,88] were also not identified in the compensatory muscle growth signature. Further, restoration of normal expression levels of hyperplasia-correlated genes such as myogenic factor MyoD2, myogenin, myogenic differentiation 1 (myod1), myogenic factor 5, and myogenic microRNAs (miR-214, miR-206-5p, miR-221-5p, miR-222, miR-133b-3p, and miR-21) provide evidence that hyperplasia is not likely contributing to the compensatory growth signature in Nile tilapia. This result agrees with Johansen and Overturf [89], and Rescan et al. [65]. Consistently, key epigenetic determinants of muscle differentiation, such as smarca4, were not upregulated, suggesting an absence of myogenic differentiation during compensatory growth. By contrast, the compensatory muscle growth signature showed upregulation of RNA methylation and modification genes. Members of the protein arginine N-methyltransferases family, such as the protein arginine methyltransferase (Prmt5), were found in the tilapia compensatory signature. Prmt5 is essential for the induction of myogenic microRNAs during differentiation [90]. Epigenetic regulators include members of the protein arginine N-methyl transferase family, including Prmt1-A, Prmt-B Prmt3, Prmt5, Prmt6, and Prmt7, were also found in the compensatory muscle growth signature of rainbow trout [65]. A similar discrepancy in the expression of the epigenetic regulators smarca4 and Prmt5 was previously reported in rainbow trout during fasting-induced recovery growth [65]. The role of chromatin-remodeling factors in the compensatory muscle growth response warrants further investigation.

The expression of genes promoting myofiber hypertrophy increased after refeeding and continued to exceed the normal expression in control fish. In agreement with the results from previous fasting-induced recovery growth transcriptomic studies [65], genes promoting ribosome biogenesis or enhancing the efficiency of the translational machinery were upregulated in compensatory muscle growth, suggesting muscle fiber hypertrophy occurs during the compensatory growth response through accretion of the necessary protein mass. Consistently, previous studies revealed a correlation between ribosome biogenesis and muscle hypertrophy [91]. The evidence supports the notion that ribosome biogenesis in skeletal muscle is an essential mechanism regulating protein synthesis and, thus, muscle mass [92]. Upregulation of heat shock proteins HSP90 and HSP70 also enhances the capacity to produce functional three-dimensional structures from nascent polypeptides. HSP90 is essential for myofibril assembly in zebrafish embryos. Further, HSP90 expression increased during muscle hypertrophy in rats and mice. Genetic ablation of Hsp70 in mice reduced myofiber size and muscle quality [93]. Furthermore, downregulation of myogenic microRNAs such as miR-1, miR-206, mir-10a, miR-139, miR-135b, miR-150, miR-185, and members from miR-30 family (miR-30a-5p, -30c -1-3p and -30c -2-3p), is associated with muscle hypertrophy. The expression levels of miR-1 were greatly increased during myogenesis and decreased during muscle hypertrophy [66,94]. In tilapia, inhibition of miR-206 was associated with an increase in body growth [39]. MicroRNA-10a, -139, -135b, -150, and -185 are anti-hypertrophic microRNAs modulating cardiac hypertrophy by targeting different biological pathways [95,96]. Overexpression of miR-30 family microRNAs enhances myogenic differentiation [97]. By contrast, microRNAs promoting muscle hypertrophy such as miR-27a-3p, miR-29c, and miR-29c, were upregulated. For instance, microRNA-27a-3p promotes muscle hypertrophy by targeting myostatin and enhancing myoblast differentiation [73]. Consistent with this result, myostatin was downregulated following refeeding. Similarly, the miR-29c was previously identified as a potent inducer of skeletal muscle hypertrophy [74]. Whereas microRNA-29a stimulates hypertrophy by targeting the PTEN/AKT/mTOR signaling pathway and inhibiting autophagy [75]. Genes promoting proteolysis/catabolic processes were significantly downregulated after refeeding. Downregulated transcripts were enriched in biological GO terms linked to negative regulation of TOR signaling and the pathways of mitophagy and autophagy. Resistance exercise-induced hypertrophy was achieved in rats by restricting autophagy-stimulated catabolism [98]. Previously reported hypertrophy-associated genes [69] (myostatin, TP53INP2, and NCoR1), which negatively affect the muscle mass [99,100,101], were also identified among downregulated genes upon refeeding. The well-known double muscling cattle breeds have mutations in the myostatin coding sequence [99]. In mammals, TP53INP2 demonstrated a significant role in autophagy [100]. Interestingly, loss of the muscle-specific NCoR1 increased the muscle mass and oxidative capacity in mice [101]. Together, our results suggest that the compensatory growth response likely results from the continuous stimulation of muscle hypertrophy, which exceeded normal levels, found in control fish. Understanding the molecular mechanisms regulating muscle degeneration and recovery will help develop better feeding regimes to enhance muscle growth and reduce production costs.

## Figures and Tables

**Table 1:** qPCR quantification revealed consistency with the RNA-Seq (R = 0.89; *p*-value 0.003). Correlation between the transcript relative expression level and body weight was performed by real-time PCR in 31 individual fish. Negative R values (−) indicates negative correlations.

Transcript Id	Annotation	Starved/Fed				Refed/Control				Correlation with Body Weight	
		RNA-Seq		qPCR		RNA-Seq		qPCR			
		log2 fc	padj	log2 fc	*p*-Value	log2 fc	padj	log2 fc	*p*-Value	R	*p*-Value
ENSONIT00000034906	F-box only protein 32	6.68	7.94 × 10^−24^	9.67	7.05 × 10^−9^	−2.36	5.2 × 10^−3^	−3.18	1.7 × 10^−2^	−0.69	9.83 × 10^−5^
ENSONIT00000080661	autophagy related 2A	7.51	1.18 × 10^−19^	3.29	1.93 × 10^−7^	N/A	N/A	−1.87	8.5 × 10^−3^	−0.66	2.2 × 10^−4^
ENSONIT00000062024	nuclear receptor corepressor 1	1.80	3.26 × 10^−2^	1.56	1.71 × 10^−4^	−3.53	4.23 × 10^−7^	−1.47	7.1 × 10^−3^	−0.40	4.2 × 10^−2^
ENSONIT00000072045	neurofilament, light polypeptide b	7.20	8.38 × 10^−14^	4.55	4.48 × 10^−3^	N/A	N/A	N/A	N/A	−0.41	3.8 × 10^−2^
ENSONIT00000022026	tumor protein p53 inducible nuclear protein 2	−3.34	2.26 × 10^−38^	−3.64	1.09 × 10^−5^	−2.67	2.98 × 10^−12^	−3.14	4.7 × 10^−5^	0.82	2.36 × 10^−7^

**Table 2:** Genes associated with skeletal muscle hypertrophy were identified in the tilapia compensatory growth response.

Transcript Id	Fold Change	*p* Value	Padj	Annotation
ENSONIT00000060950	3.12	2.00 × 10^−5^	2.88 × 10^−4^	Transcription Factor Jun-B (AP-1)
ENSONIT00000039894	2.31	6.07 × 10^−3^	3.59 × 10^−2^	Calpastatin
ENSONIT00000016158	−2.03	6.12 × 10^−6^	1.03 × 10^−4^	Nuclear receptor corepressor 1
ENSONIT00000055439	−3.07	2.30 × 10^−3^	1.64 × 10^−2^	Diacylglycerol O-acyltransferase 1
ENSONIT00000076842	−3.53	6.51 × 10^−8^	1.92 × 10^−6^	Bradykinin receptor B2
ENSONIT00000045589	−3.61	1.08 × 10^−3^	8.78 × 10^−3^	Inhibin beta B chain
ENSONIT00000048569	−3.76	5.74 × 10^−6^	9.70 × 10^−5^	Myostatin
ENSONIT00000022026	−6.36	2.2 × 10^−14^	2.98 × 10^−12^	Tumor protein p53 inducible nuclear protein 2
ENSONIT00000062024	−11.55	1.19 × 10^−8^	4.23 × 10^−7^	Nuclear receptor corepressor 1

## Data Availability

The datasets generated and/or analyzed during the current study are available in the Sequence Read Archive (SRA) repository, NCBI BioProject PRJNA842822.

## Supplementary Materials

The following supporting information can be downloaded at: https://www.mdpi.com/article/10.3390/cells11162504/s1. Supplementary Table S1: Differential transcripts (mRNAs, lncRNAs & microRNAs) between starved/fed, refed/control, and refed/starved groups (log2FC > |1|, FDR < 0.05); Table S2: Differential microRNAs reciprocally correlated in expression with the differential target transcripts (mRNAs & lncRNAs); Table S3: protein-coding transcripts and lncRNAs exhibiting expression correlation and harboring the same microRNA binding sites
